# Effects of Continuous Intake of Rosemary Extracts on Mental Health in Working Generation Healthy Japanese Men: Post-Hoc Testing of a Randomized Controlled Trial

**DOI:** 10.3390/nu12113551

**Published:** 2020-11-20

**Authors:** Risa Araki, Kazunori Sasaki, Hiroyuki Onda, Syunsuke Nakamura, Masahiro Kassai, Toshiyuki Kaneko, Hiroko Isoda, Koichi Hashimoto

**Affiliations:** 1 Department of Clinical and Translational Research Methodology, Faculty of Medicine, University of Tsukuba, 1-1-1 Tennodai, Tsukuba 305-8575, Japan; risa.araki@md.tsukuba.ac.jp; 2 R&D Center for Tailor-Made QOL, University of Tsukuba, 1-2 Kasuga, Tsukuba 305-8550, Japan; isoda.hiroko.ga@u.tsukuba.ac.jp; 3 Open Innovation Laboratory for Food and Medicinal Resource Engineering (Food-Med OIL), AIST-University of Tsukuba, 1-1-1 Higashi, Tsukuba 305-8565, Japan; sasaki-kazu@aist.go.jp; 4 Faculty of Pure and Applied Sciences, University of Tsukuba, 1-1-1 Tennodai, Tsukuba 305-8571, Japan; 5 Alliance for Research on the Mediterranean and North Africa (ARENA), University of Tsukuba, 1-1-1 Tennodai, Tsukuba 305-8577, Japan; 6 S&B FOODS INC., 38-8 Miyamoto-cho, Itabashi-ku, Tokyo 174-8651, Japan; hiroyuki_onda@sbfoods.co.jp (H.O.); shunsuke_nakamura@sbfoods.co.jp (S.N.); masahiro_kassai@sbfoods.co.jp (M.K.); 7 Tokyo Skytree Station Medical Clinic, 3-33-13 Sumida-ku, Tokyo 131-0033, Japan; kaneko@skytree-clinic.jp; 8 Faculty of Life and Environmental Sciences, University of Tsukuba, 1-1-1 Tennodai, Tsukuba 305-8577, Japan

**Keywords:** rosemary extracts, dietary intervention, mood states, fatigue, cognitive function, mental health, clinical trial

## Abstract

We previously performed a 4 week interventional trial that suggested that continuous intake of rosemary extract improves the mood states, fatigue, and cognitive function of working generation healthy adult Japanese men. However, the severity of depression in participants in our previous study was relatively mild. Therefore, in the present study, a post-hoc analysis of our previous study was conducted, limited to participants whose total mood disturbance (TMD) scores, which indicate greater mood disturbance, were above the median at baseline, to evaluate whether rosemary extract was effective for individuals with poor mental health. Following the intervention, the scores of TMD and “Confusion-Bewilderment” were significantly decreased (both *p* < 0.05), and scores of “Vigor-Activity” were significantly increased in the rosemary group (*n* = 8) compared with those in the control group (*n* = 13; *p* < 0.01). When comparing the scores from pre- and post-intervention, significant improvements in “Tension-Anxiety”, “Vigor-Activity”, “Fatigue on awakening”, “Daytime sleepiness”, and “Psychomotor speed” were observed in the rosemary group only (all *p* < 0.05). Based on these results, it was expected that rosemary extracts were effective for improving the mental energy and sleep quality of work-age men with poor mental health.

## 1. Introduction 

Work-related stress has been suggested to lead to poor mental health [1] and is associated with poorer neurocognitive performance in middle-aged adults [2]. Work-related stress can induce poor productivity, human errors [3], high levels of absenteeism [4], and chronic fatigue [5], partly underpinned by oxidative stress [6]. These factors lead to a reduction in the quality of life and economic loss [7]. Thus, early prevention and improvement of work-related stress are critical unmet needs.

Rosemary is an aromatic herb rich in antioxidant compounds such as rosmarinic acid [8]. Rosemary has been used as a dietary spice and traditional medicine [9]. In animal experiments, administration of rosemary extracts and rosmarinic acid has been associated with anti-depressant and neuroprotective effects [10,11], as well as improvements in learning and memory [12,13]. Although clinical evidence for the beneficial effects of rosemary extracts is lacking, improvements in memory performance, anxiety, depression, and sleep quality in university students following continuous intake of dried powdered aerial parts of rosemary for 1 month have been reported [14]. Furthermore, improvements in delayed word recall (indicative of short-term memory) were observed in the subgroup analysis limited to healthy subjects aged 40–62 years who continuously ingested combined extracts of sage, rosemary, and Melissa rich in rosmarinic acid for 2 weeks [15]. Whereas the effectiveness of rosemary extract for improving mood states and cognitive function had not been clearly shown in several short-term studies [16,17]. 

Based on these reports, we previously conducted a 4 week randomized controlled trial, which suggested that continuous intake of rosemary extracts improved mood states, fatigue, and cognitive function of healthy adult Japanese men [18]. Individuals with chronic fatigue have been considered to be more likely to develop depression [19]. Thus, we recruited individuals who felt fatigue chronically in our previous study; however, the severity of depression in our participants was relatively mild [18].

Rosemary extracts are derived from natural sources and are easily ingested. We previously confirmed that our rosemary extract has no side effects and the frequencies of adverse events in the rosemary group were not different from that in the control group, based on their daily physical condition records during the intervention period [18]. We hypothesized that the beneficial effects of rosemary extracts on mental health that we previously observed could also be obtained in individuals experiencing emotional and mood disturbances, and would contribute to the development of a safe and easily obtainable therapeutic to promote mental health in working-age men. 

The total mood disturbance (TMD) scores indicate greater mood disturbance and are calculated by using the scores of six mood clusters on the Profile of Mood States Second Edition (POMS2). In particular, subtracting the score of one positive mood cluster from the summing of the scores of five negative mood clusters [20]. In this study, we performed a post-hoc analysis of our previous study limited to the participants whose TMD scores was above the median at baseline. 

## 2. Materials and Methods 

### 2.1. Study Protocol and Participants

In our previous double-blind, placebo-controlled, parallel-group comparative study, we recruited healthy Japanese men aged 20–64 years who felt fatigue on a daily basis. A briefing session for this study was conducted. Written informed consent was obtained from 75 candidates before the screening test. 

The exclusion criteria were as follows: currently being treated for chronic diseases; may experience allergies related to the test products; medical history of mental or metabolic disorders; night or shift workers; current smokers; having highly irregular lifestyles; having an unbalanced diet; taking medications or regularly consuming supplements (including “food for specified health use” and “foods with function claims”) that affect stress, cognitive function, and sleep; having participated within 3 months or planned to participate in other clinical studies during the period of our study; or recognized as unsuitable for this study by the principal investigator.

In this study, the Beck Depression Inventory-II (BDI-II), and questionnaire about health conditions and lifestyles were applied as a screening tool. Of the 75 candidates, 42 healthy individuals with a BDI-II score less than 21 points and those confirmed to be eligible were enrolled in the intervention and were randomly divided into the rosemary group (*n* = 21) and control group (*n* = 21) by the data coordinator. For assignment, a simple randomization method using a computer-generated number sequence was employed. The allocation sequence was concealed from participants and other research staff during the intervention. After fixing the data, the assignment was disclosed to research staff including the data analyst.

During the 4 week intervention, participants were instructed as follows: take 1 g of the test food dissolved in 100 cc of hot or cold water after breakfast or at midmorning; record and send a diary on the status of test meal intake and physical conditions daily via the online system; maintain usual diet and lifestyle during the study period. Research staff monitored daily records and contacted participants by e-mail or telephone if necessary. 

The protocol of the present study was performed in accordance with the Declaration of Helsinki and was approved by the Nihonbashi Egawa Clinic Clinical Research Ethics Committee (approval number: R01-116). This study was registered with the University Hospital Medical Information Network Clinical Trials Registry System (https://www.umin.ac.jp/ctr) as UMIN000036762. Subsequently, survey and data collection were performed between 27 May and 5 July 2019 at the Tokyo Skytree Station Medical Clinic (Tokyo, Japan) by research staff of HUMA R&D Co., Ltd. (Tokyo, Japan). 

**Figure 1:**
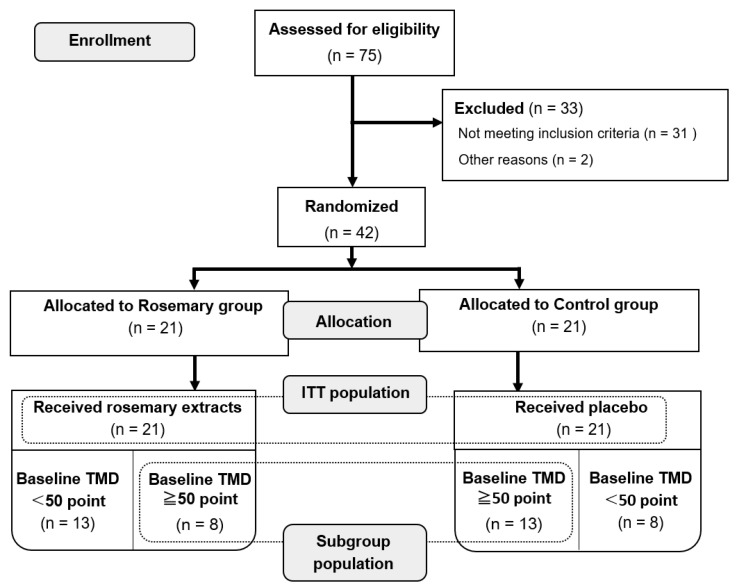
Diagram illustrating the selection of participants for analysis. TMD: total mood disturbance; ITT: intension to treat.

In our previous study, the TMD score of participants (*n* = 42) was 50.0 (46.0–55.0) (median (interquartile range (IQR))) points. In this study, we used the data of the subgroup population (rosemary group: *n* = 8, control group: *n* = 13) with TMD scores above the median (50 points) at baseline (Figure 1).

### 2.2. Test Foods

The rosemary powder for the rosemary group was prepared by mixing rosemary extract obtained by hot-water extraction, trehalose, and dextrin at a ratio of 6:47:47. The placebo powder for the control group was prepared by mixing trehalose and dextrin at a ratio of 50:50. Mixtures were pulverized using the spray-drying method at T. HASEGAWA Co., Ltd., which acquired the International Organization for Standardization (ISO) 22000 certification, and were filled into aluminum bags weighing 1 g each. The rosemary powder provided 8 mg of rosmarinic acid, the main component, daily. Although 1,8-cineole, carnosic acid, and luteolin have been reported to be in rosemary essential oils [21] and ethanol extracts [10,22], these were not detected in our rosemary hot-water extract.

### 2.3. Survey Questionnaires

In this study, we used the POMS2 as a marker of mood states in the last 7 days, including the measurement day; Cognitrax as a marker of stress-related cognitive function; and visual analog scale (VAS) as a subjective marker of current quality of sleep and fatigue. All survey questionnaires were administered at baseline and at the end of the intervention. POMS2 and Cognitrax scores at the end of the intervention were the primary endpoints. VAS was the secondary endpoint. On each measurement day, the surveys were conducted in the order POMS, VAS, and Cognitrax, and the required times were about 10, 5, and 60 min, respectively.

The Japanese translation of the full-length version of the POMS2, consisting of 65 self-report items using a 5-point scale from “not-at-all” to “extremely” [20], was used in this study. Based on the obtained answers, the TMD score that comprehensively represented a negative mood state, and the scores of seven subscales encompassing “Anger-Hostility”, “Confusion-Bewilderment”, “Depression-Dejection”, “Fatigue-Inertia”, “Tension-Anxiety”, “Vigor-Activity”, and “Friendliness” were calculated as T-scores standardized by age and sex.

We used the Cognitrax test based on the Central Nervous System (CNS) Vital Signs test [23], which is implemented by combining multiple electronic psychological tests [24,25]. In this study, verbal memory, visual memory, finger tapping, symbol digit coding (SDC), Stroop, attention shifting, 10-type, continuous processing, facial expression recognition, logical thinking, and 4-part continuous processing tests were conducted. We obtained “standardized score” data converted with the average value of the same age (being 100) and standard deviation of 15 for “Neurocognitive Index (NCI)” and 11 cognitive function areas of “Composite memory”, “Verbal memory”, “Visual memory”, “Psychomotor speed”, “Reaction time”, “Complex attention”, “Cognitive flexibility”, “Processing speed”, “Executive function”, “Simple attention”, and “Motor speed.”

A VAS was used to evaluate six items: “Clear thinking”, “Attentiveness”, “Concentration”, “Fatigue on awakening”, “Sleepiness on awakening”, and “Daytime sleepiness.” Specifically, for each parameter, a vertical line was drawn within a 100 mm line segment according to the degree to which each person felt and measured the distance from the left end to the vertical line to produce a score. For “Clear thinking”, “Attention”, and “Concentration”, the left and right ends were set as “not at all” and “at the highest”, respectively, whereas those for “Fatigue on awakening”, “Sleepiness on awakening”, and “Daytime sleepiness” were set at left-right reversal. For all items, the right end and higher scores reflected better conditions.

### 2.4. Statistical Analysis

For descriptive statistics, unless otherwise specified, the median with interquartile range (IQR) are shown. Due to the small sample size, we used nonparametric analyses in this study. In particular, a Mann–Whitney U test was used to compare the scores of each evaluation item between two groups at the start. Within-group differences were compared using the Wilcoxon signed-rank test. The scores at the end of the intervention and changes in these items were analyzed by the Quade test with each initial value as a covariate. Fisher’s exact test was used to compare the incidence of adverse events during the intervention between groups. IBM SPSS Statistics 25 (IBM Japan, Tokyo, Japan) was used for all statistical analyses. *p* < 0.05 was set as the level of statistical significance (two-sided).

## 3. Results

### 3.1. Baseline Characteristics

At the pre-intervention, the age of the subgroup population (*n* = 21) was 41.0 (31.0–48.0) (median (IQR)) years, BDI score was 11.0 (9.0–13.0) points, and sleeping duration was 7.0 (6.5–7.0) h. No significant differences were observed in these parameters at pre-intervention between the rosemary group (*n* = 8) and control group (*n* = 13) (data not shown). The number of participants with a BDI-II score of 14–19 points (indicative of mild depression) [26] was four (*n* = 2 in each group, *p* = 0.618).

### 3.2. POMS T-Scores Pre- and Post-Intervention

As shown in Table 1, POMS T-scores for all parameters at week 0 were not significantly different between groups. After the intervention, T-scores of “Confusion-Bewilderment” (*p* < 0.05), TMD (*p* = 0.051), “Depression-Dejection” (*p* = 0.058), and “Fatigue-Inertia” (*p* = 0.076) were significantly lower or tended to be lower in the rosemary group compared with the control group. In addition, T-score of “Vigor-Activity” was significantly higher in the rosemary group than in the control group (*p* < 0.01). On comparing the scores at pre- and post-intervention, significant improvements in “Tension-Anxiety” and “Vigor-Activity” were observed but only in the rosemary group (both *p* < 0.05).

### 3.3. Changes in POMS T-Scores during the Intervention

**Figure 2:**
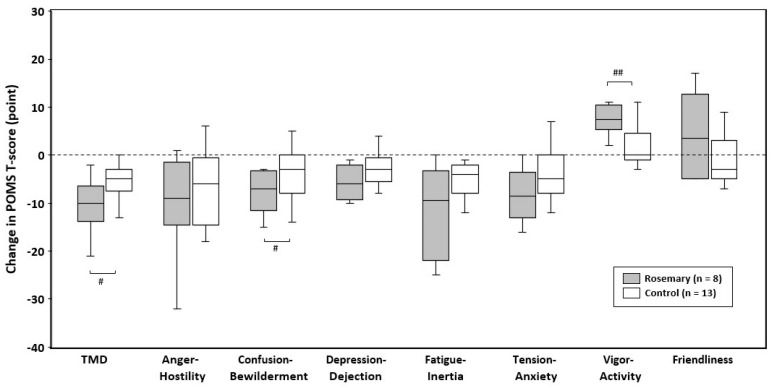
Changes in POMS T-scores during the intervention. Box-and-whisker plot representing the median (line within the box), IQR (length of the box), and the maximum and minimum values (whiskers above and below the box). Significant differences between groups were based on Quade test with each baseline value as covariate: ^#^ *p* < 0.05, ^##^ *p* < 0.01. TMD: total mood disturbance.

As shown in Figure 2, the rosemary group exhibited negative changes in T-scores of TMD, “Anger-Hostility”, “Confusion-Bewilderment”, “Depression-Dejection”, “Fatigue-Inertia”, and “Tension-Anxiety.” In contrast, positive changes were noted in T-scores of “Vigor-Activity” and “Friendliness.” TMD and “Confusion-Bewilderment” T-scores were significantly decreased to a greater degree (both *p* < 0.05) and “Vigor-Activity” T-score was significantly increased to a greater degree in the rosemary group compared to those in the control group (*p* < 0.01).

### 3.4. VAS Scores Pre- and Post-Intervention

All parameters at week 0 were not significantly different between groups. At the end of the study, VAS scores of all parameters were higher in the rosemary group than in the control group, and of those, “Daytime sleepiness” scores were significantly different between the two groups (both *p* < 0.05). In the comparison between pre- and post-intervention scores for each group, “Fatigue on awakening” and “Daytime sleepiness” were significantly changed in the rosemary group, and “Attentiveness” was significantly changed in the placebo group (all *p* < 0.05) (Table 2).

### 3.5. Changes in VAS Scores during the Intervention

**Figure 3:**
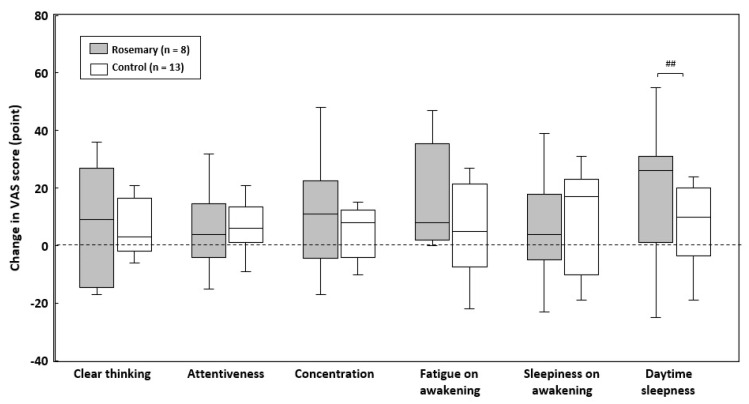
Changes in VAS scores during the intervention. Box-and-whisker plot representing the median (line within the box), the IQR (length of the box), and the maximum and minimum values (whiskers above and below the box). Indicates significance between groups based on the Quade test with each baseline value as covariate: ^##^ *p* < 0.01. VAS: visual analog scale.

Elevation in VAS scores of “Clear thinking”, “Concentration”, “Fatigue on awakening”, and “Daytime sleepiness” was higher in the rosemary group than in the control group. Of those, significant differences in “Daytime sleepiness” scores were observed between groups (*p* < 0.01) (Figure 3).

### 3.6. NCI and Cognitive Domain Scores Calculated in Cognitrax Pre- and Post-Intervention

At pre-intervention, no parameters were significantly different between groups. In the rosemary group only, there was a significant increase in “Psychomotor speed” compared with baseline value (*p* < 0.05) (Table 3).

## 4. Discussion

In the present study, we performed an analysis limited to participants that scored high in TMD scores at baseline in our previous study [18]. Changes in the TMD, “Confusion-Bewilderment”, “Vigor-Activity”, and “Daytime sleepiness” were more favorable in the rosemary group compared with the placebo group. Additionally, significant improvements from baseline values in “Tension-Anxiety”, “Vigor-Activity”, “Fatigue on awakening”, “Daytime sleepiness”, and “Psychomotor speed” were observed in the rosemary group only. Therefore, increases in mental energy and sleep quality were expected because of the effectiveness of our rosemary extract. These results support our previous findings [18] and agree with the results of Nematolahi et al., who demonstrated improvements in anxiety, depression, and sleep quality in university students after 1 month of rosemary powder intake [14].

A positive association between mental energy and sleep quality has also been confirmed in several studies [27,28]. In addition, Takeuchi et al. reported that sleep quality showed negative correlations with “Confusion-Bewilderment” and “Tension-Anxiety” [28]. The activity of the autonomic nervous system (ANS) is considered one of the factors related to emotional reactions [29].

Rosmarinic acid, the main component of our rosemary extract, enhanced the action of cholinergic transmission that contributed to the regulation of the ANS [30]. The ANS is also known to be linked with cognitive function [31]. The “Psychomotor speed”, which showed favorable changes only in the rosemary group, reflects the relationship between cognitive abilities and physical movements [32]. Changes in the “Psychomotor speed” of the rosemary group might be related to ANS activation by the rosemary extract.

Reactive oxygen species (ROS) reduction in the brain has been suggested to lead to cognitive impairment reduction [33]. The hippocampus and prefrontal cortex are involved in memory and learning [34], and the prefrontal cortex is implicated in emotion generation [35]. Rosmarinic acid protects neuronal cells against cellular stress injury [10,36], suppresses intracellular increases in ROS [37], and exerts protective effects in dopaminergic neurons by promoting the antioxidant enzyme superoxide dismutase expression [38]. Administration of rosmarinic acid reduced blood corticosterone levels in mice subjected to tail suspension stress and exerted antioxidant and neuroprotective effects in the hippocampus and prefrontal cortex [11].

Although the results did not reach statistical significance, the scores for “Depression-Dejection”, and “Fatigue-Inertia” in the rosemary group showed a trend of being lower than those in the control group. Rosmarinic acid exerts antidepressant-like effects via proliferation of neoplastic cells in the dentate gyrus of the hippocampus [39]. It has been reported that rosmarinic acid reduces oxidative stress and apoptosis in the prefrontal cortex and suppresses symptoms of anxiety and depression [40]. Fatigue activates the hypothalamic–pituitary–adrenal axis and increases blood levels of glucocorticoids [41]. Based on these findings, we presumed that neuroprotective effects of rosmarinic acid through anti-oxidation also occurred in the hippocampus and prefrontal cortex in this study.

Rosemary essential oils and extracts obtained by ethanol extraction include carnosic acid, luteolin, and the aroma component 1.8-cineole [10,21,22]. It is believed these ingredients also improve emotion and sleep quality [42,43]; however, these components were not detected in our rosemary hot-water extract. Therefore, the effects of the rosemary extract observed in this study were probably due to the main component, rosmarinic acid, rather than lipophilic or other components.

In this study, we demonstrated that continuous intake of rosemary extracts led to improvements in vigor, sleep quality, and cognitive function. It has been suggested that these are closely related to the frontal lobe [34,35,44]. We did not confirm frontal lobe activity in the current study; however, it is possibility that our rosemary extract had beneficial effects for the frontal lobe function in participants with relatively high TMD scores. A reduction in “Confusion-Bewilderment” score and elevation in “Vigor-Activity” scores were parameters that were overlooked in the analysis that included individuals with low TMD scores. These measures, which are related to improvements in work efficiency, may have been favorably altered due to a decrease in work-related stress. The possibility was also considered that the effects of rosemary extract that were expected in this study may be applicable for the self-medication of people who have mood disorders but are reluctant to receive treatment.

However, we were unable to confirm whether high TMD scores were underpinned by work-related stress in this study. Considering a T-score of 50 points is equivalent to the average for the Japanese population, the severity of mood disturbance of participants in this study was not very high, as their median TMD T-score was around 55. The number of participants was very small due to the post-hoc nature of the analysis, and they all lived in a specific area. We focused on men aged between 20–64 years of the working generation, and the effects of rosemary extract in younger and middle-aged people may have differed. Furthermore, because the sample size of the current study was extremely small, the effects of potential confounders, including other factors, could not be investigated, and the duration of this intervention was very short. The mechanisms of the effects of our rosemary extract observed in this study have not been fully revealed. Therefore, it will be necessary to conduct more long-term follow-up studies on a larger population with other backgrounds to examine the benefits of our rosemary extract on mental health while adding evaluation items and considering confounding factors and generalization.

## 5. Conclusions

In this post-hoc analysis, we revealed that the continuous intake of rosemary extract might be associated with improvements in the mental energy and sleep quality of individuals with relatively high TMD scores, underscoring the potential beneficial effects of rosemary extracts on cognitive function.

## Figures and Tables

**Table 1:** Profile of Mood States (POMS) T-scores before and after the intervention.

T-Score (Point)		Rosemary (*n* = 8)	Control (*n* = 13)	*p* ^b^
TMD	0 week	57.0 (53.5–59.5) ^a^	54.0 (54.0–56.0)	0.374
	4 week	47.5 (45.0–50.5) *	50.0 (48.0–51.0) **	0.051 ^c^
Anger- Hostility	0 week	53.0 (47.0–60.0)	52.0 (49.0–61.0)	0.804
	4 week	45.0 (43.0–47.0) *	48.0 (44.0–53.0) *	0.483 ^c^
Confusion- Bewilderment	0 week	55.5 (46.5–62.0)	55.0 (49.0–59.0)	0.916
	4 week	46.5 (40.0–54.0) *	50.0 (47.0–55.0) *	0.040 ^c^
Depression- Dejection	0 week	54.0 (47.5–57.0)	50.0 (49.0–53.0)	0.456
	4 week	46.5 (41.5–53.0) *	46.0 (44.0–49.0) *	0.058 ^c^
Fatigue- Inertia	0 week	61.0 (58.0–66.0)	57.0 (55.0–59.0)	0.053
	4 week	50.0 (45.0–53.0) *	55.0 (46.0–55.0) **	0.076 ^c^
Tension- Anxiety	0 week	57.0 (56.5–60.0)	56.0 (53.0–61.0)	0.645
	4 week	48.0 (44.0–57.0) *	53.0 (49.0–57.0)	0.171 ^c^
Vigor- Activity	0 week	46.0 (37.5–47.0)	44.0 (39.0–47.0)	0.972
	4 week	53.0 (47.5–56.5) *	44.0 (41.0–52.0)	0.010 ^c^
Friendliness	0 week	51.0 (33.0–54.5)	47.0 (42.0–50.0)	0.645
	4 week	49.5 (43.5–55.5)	45.0 (40.0–47.0)	0.307 ^c^

^a^ median (interquartile range (IQR)). *p*-values were computed via ^b^ Mann–Whitney U test and ^c^ Quade test with each baseline value as covariate. * *p* < 0.05 and ** *p* < 0.01 vs. 0 week based on Wilcoxon signed-rank test. TMD: total mood disturbance.

**Table 2:** Visual analog scale (VAS) scores pre- and post-intervention.

Score (Point)		Rosemary (*n* = 8)	Control (*n* = 13)	*p* ^b^
Clear thinking	0 week	42.0 (25.5–59.0) ^a^	37.0 (31.0–43.0)	0.645
	4 week	46.0 (43.5–55.5)	45.0 (33.0–50.0)	0.351 ^c^
Attentiveness	0 week	41.5 (26.5–55.5)	35.0 (29.0–44.0)	0.456
	4 week	44.0 (40.5–54.0)	40.0 (33.0–50.0) *	0.441 ^c^
Concentration	0 week	42.5 (20.0–51.5)	35.0 (28.0–55.0)	0.916
	4 week	50.5 (45.5–55.5)	49.0 (33.0–49.0)	0.127 ^c^
Fatigue on awakening	0 week	25.5 (20.5–33.0)	28.0 (24.0–37.0)	0.336
	4 week	48.5 (32.0–57.0) *	32.0 (30.0–51.0)	0.463 ^c^
Sleepiness on awakening	0 week	34.0 (30.5–51.5)	31.0 (21.0–39.0)	0.301
	4 week	47.0 (38.5–50.0)	43.0 (30.0–51.0)	0.840 ^c^
Daytime sleepiness	0 week	40.0 (19.5–50.0)	31.0 (23.0–38.0)	0.645
	4 week	57.5 (50.5–69.0) *	45.0 (25.0–51.0)	0.019 ^c^

^a^ median (IQR). *p*-values were computed via ^b^ Mann–Whitney U test and ^c^ Quade test with each baseline value as covariate. * *p* < 0.05 vs. 0 week based on Wilcoxon signed-rank test. VAS: visual analog scale.

**Table 3:** Neurocognitive index (NCI) and cognitive domain scores calculated in Cognitrax pre- and post-intervention.

T-Score (Point)		Rosemary (*n* = 8)	Control (*n* = 13)	*p* ^b^
NCI	0 week	107.0 (101.0–110.0) ^a^	108.0 (102.0–113.0)	0.547
	4 week	107.0 (99.5–114.0)	107.0 (94.0–114.0)	0.195 ^c^
Composite memory	0 week	103.5 (95.5–113.5)	114.0 (107.0–119.0)	0.121
	4 week	96.0 (92.5–103.0)	109.0 (94.0–112.0)	0.584 ^c^
Verbal memory	0 week	103.5 (95.5–111.3)	115.0 (106.0–125.0)	0.053
	4 week	108.0 (95.3–114.3)	109.0 (96.0–123.0)	0.575 ^c^
Visual memory	0 week	106.0 (96.0–111.5)	109.0 (100.0–113.0)	0.645
	4 week	92.5 (86.5–96.0) *	104.0 (87.0–112.0)	0.237 ^c^
Psychomotor speed	0 week	111.5 (102.0–126.0)	116.0 (106.0–126.0)	0.697
	4 week	126.0 (100.0–133.0) *	123.0 (107.0–134.0)	0.442 ^c^
Reaction time	0 week	95.5 (90.0–100.5)	99.0 (88.0–103.0)	0.645
	4 week	98.0 (93.5–100.5)	96.0 (91.0–102.0)	0.443 ^c^
Complex attention	0 week	107.5 (105.0–112.5)	108.0 (98.0–114.0)	0.697
	4 week	113.5 (98.0–114.5)	108.0 (88.0–113.0)	0.394 ^c^
Cognitive flexibility	0 week	108.5 (104.0–114.0)	109.0 (104.0–111.0)	0.804
	4 week	112.5 (108.0–118.0)	110.0 (91.0–113.0)	0.265 ^c^
Processing speed	0 week	111.5 (104.0–117.5)	117.0 (111.0–124.0)	0.301
	4 week	116.0 (108.0–123.5)	121.0 (113.0–132.0)	0.172 ^c^
Executive function	0 week	109.0 (103.5–114.5)	109.0 (104.0–113.0)	0.972
	4 week	113.0 (108.5–118.5)	109.0 (94.0–113.0)	0.127 ^c^
Simple attention	0 week	106.0 (89.5–107.0)	106.0 (85.0–107.0)	0.645
	4 week	106.5 (106.0–107.0)	106.0 (82.0–107.0)	0.381 ^c^
Motor speed	0 week	113.5 (91.0–125.5)	105.0 (99.0–122.0)	0.697
	4 week	125.5 (92.5–131.0)	109.0 (99.0–117.0)	0.333 ^c^

^a^ median (IQR). *p*-values were computed via ^b^ Mann–Whitney U test and ^c^ Quade test with each baseline value as covariate. * *p* < 0.05 vs. 0 week based on Wilcoxon signed-rank test. NCI: neurocognitive index.
